# Complication rare et grave de la rachianesthésie: la méningite bactérienne (à propos d’un cas et revue de la littérature)

**DOI:** 10.11604/pamj.2017.27.158.12348

**Published:** 2017-06-30

**Authors:** Naoufal Chouaib, Said Jidane, Mostafa Rafai, Ahmed Belkouch, Saad Zidouh, Lahcen Belyamani

**Affiliations:** 1 Service des Urgences Médico-Chirurgicales, Hôpital Militaire Mohamed V, Faculté de Médecine et de Pharmacie, Rabat, Maroc

**Keywords:** Méningite bactérienne, rachianesthésie, nosocomiale, complication grave, asepsie, Bacterial meningitis, spinal anesthesia, nosocomial, serious complication, asepsis

## Abstract

La rachianesthésie (RA) est la première anesthésie locorégionale. Elle comporte des effets secondaires et des risques qu'il faut pouvoir éviter, prévenir ou traiter précocement. C'est le cas d'une patiente opérée sous rachianesthésie qui a présenté quelques jours après l'interventionde céphalées intenses associées à des nausées et des vomissements évoluant dans un contexte de fièvre. La ponction lombaire mettait en évidence un liquide trouble avec la présence de cocci gram + à l'examen direct, ce qui a permis de poser le diagnostic d'une méningite bactérienne et dont l'évolution a été favorable après antibiothérapie.

## Abstract (en)

Spinal anesthesia (SA) is the first locoregional anesthesia. It can cause side effects and carry risks that need to be avoided, prevented or treated early. We here report the case of a female patient operated under spinal anesthesia who had intense headache associated with nausea and vomiting evolving in the context of fever within a few days after surgery

Lumbar puncture showed cloudy liquid revealing Gram + cocci on direct examination. This allowed the diagnosis of bacterial meningitis. Patient’s evolution was favorable after antibiotic therapy.

## Introduction

La rachianesthésie (RA) est la première anesthésie locorégionale décrite il y a plus de cent ans [1]. Bien qu'ayant connue la « concurrence » de la péridurale et de l'anesthésie générale, la RA représente environ 40% des anesthésies locorégionales réalisées en France [2]. L'utilisation fréquente de cette technique réside dans sa simplicité et dans l'efficacité du blocage neuronal obtenu. En effet, la RA est habituellement sûre et performante, particulièrement chez la parturiente, chez l'insuffisant respiratoire, chez les patients à « estomac plein »,… Mais, la rachianesthésie comporte des effets secondaires et des risques qu'il faut pouvoir éviter, prévenir ou traiter précocement [1]. Nous rapportons le cas d'une patiente opérée sous rachianesthésie qui a présenté quelques jours après l'intervention une méningite bactérienne et dont l'évolution a été favorable après traitement.

## Patient et observation

Une femme de 52ans, sans antécédents notables, qui a présenté une lithiaseurétérale et dont l'indication de l'extraction du calcul, sous rachianesthésieparurétéroscopie avec montée d'une sonde double J a été posée.Cinq jours après l'intervention, la patiente se plaignait de céphalées intenses associées à des nausées et des vomissements évoluant dans un contexte de fièvre chiffrée à 39.5°c faisant craindre une méningite iatrogène. A l'admission aux urgences, la patiente présentait un syndrome méningé fébrileisolé. Le bilan biologique a montré un syndrome inflammatoire avec une hyperleucocytose à 13.600/ml et une CRP à 182mg/l. La ponction lombaire mettait en évidence un liquide trouble(GB=1600GB/microlitres)avec la présence de cocci gram + à l'examen direct, une normoglycorachie à 0.63g/l pour une glycémie concomitante à 1.65g/l avec une hyperproteinorachie à 2.96g/l. Compte tenu du caractère nosocomial de cette infection, une antibiothérapie probabiliste à large spectre était débutée àbase de vancomycine (60mg/kg/jr) et de ceftriaxone (100mg/kg/jr). Le mêmetraitement était continué et permettait d'obtenir une apyrexie en 72h avec amélioration de l'état générale. La CRP est passée à 65mg/l et les globules blancs ont régressée à 9.400/ml. La patiente a quitté l'hôpital après 12 jours d'hospitalisation sans séquelles.

## Discussion

La méningite est la complication infectieuse la plus grave de la rachianesthésie. De façon anormale sa fréquence semble être en augmentation depuis plusieurs années. En 1981, dans une revue incluant plus de 65 000 rachianesthésies, seules 3 méningites étaient rapportées. En 2004, Moen et al. [3] rapportent 32 cas de méningites sur un collectif scandinave de 1 260 000 rachianesthésies sur une période de 10 ans. Les publications les plus récentes évoquent une incidence de méningite après rachianesthésie comprise entre 3,7 et 7,2 pour 100 000 [4]. La particularité de ces méningites survenant au décours d'une rachianesthésie (ou d'une anesthésie péridurale) tient aux germes habituellement identifiés. En effet, les pathogènes le plus souvent mis en cause sont différents de ceux classiquement responsables de méningites communautaires ou postopératoires. Les germes les plus fréquemment retrouvés sont habituellement des cocci à Gram positif, essentiellement des staphylocoques ou des streptocoques alpha'hémolytiques, le plus souvent du type salivarius. Ces germes sont habituellement originaires soit de la peau du patient, soit de la flore commensale de la salive des personnels au contact du patient lors de la réalisation de la ponction et qui ne portent pas de masque de protection [1]. Il s'agit donc (presque) toujours d'une faute d'asepsie: mauvaise désinfection cutanée ou masque facial absent ou défectueux. La revue de Baer et al. [5] confirme que la majorité des germes retrouvés dans ces méningites après ponctions rachidiennes sont soit des streptocoques, dont l'espèce la plus fréquente est S. salivarius, ou viri dans, soit des staphylocoques. Dans notre cas, l'examen direct a montré une cocci gram + mais la culture n'a pas pu isoler de germes. Ces germes témoignent de précautions d'asepsie insuffisantes, mettant directement en cause la responsabilité de l'opérateur [1].

Les méningites post rachianesthésie sont des complications graves, capables d'engager le pronostic vital des patients. Les germes de ce type bien particulièrement des méningites proviennent le plus souvent de l'oropharynx de l'opérateur et sont inoculés lors du geste au point de ponction de la rachi-anesthesie par l'intermédiaire de gouttelettes de Pflügge [6]. Schématiquement les mécanismes de contamination du LCR sont doubles: inoculation directe à partir de la peau (staphylococcus aureus,staphyloccoccus epidermitis et streptococcus salivarius),du nasopharynx de l'opérateur via les goutelettes de flugge(streptococcus salivaruis)ou du sang lors d'une bactériémie concomitante; Contamination secondaire du cathéter selon deux modes: externe(à partir du revêtement cutané ou d'un foyer infectieux de proximité)ou interne par voie hématogène lors d'une septicémie ou beaucoup plus rarement d'une bactériémie [7]. L'antibiothérapie des méningites nosocomiales est relativement bien codifiée mais concernent essentiellement les méningites après intervention neurochirurgicales. Sur le plan bactériologique, les staphylocoques représentent 50 à 80% de ce type de méningites [8]. Le traitement des infections du système nerveux central nécessite des concentrations efficaces de l'antibiotique au niveau du cite de l'infection avec une bonne diffusion à travers la barrière hémato-encéphalique [7]. Vu la mauvaisediffusion de la vancomycine vers le LCR, l'augmentation de la dose de la vancomycine avec l'association a la ceftriaxone s'est avérée efficace dans notre cas. La survenue des cas de méningites iatrogènes et leur morbidités doivent inciter à la plus rigueur tant sur le plan du respect des contre-indications, en particulier infectieuses, que celui des techniques de rachianesthésie. C'est un diagnostic à ne pas négliger même devant un tableau atypique. Le meilleur traitement repose essentiellement sur la prévention et le respect des règles d'asepsie stricte: préparation chirurgicale de l'opérateur et du patient; Introduction de l'extrémité du cathéter sans contact avec les mains stériles de l'opérateur (technique du non touch) lors de la pose de l'anesthésie péridurale; Ouverture du cathéter sous pansement rigoureusement occlusif [7].

## Conclusion

Les méningites post rachianesthésie sont des complications graves, capables d'engager le pronostic vital des patients.Leurs diagnostic et prises en charges sont difficiles du fait de leurs tableaux atypiques d'où l'intérêt de ne pas hésiter de faire une ponction lombaire en cas de suspicion en post rachianesthésie. La prévention passe par la sensibilisation des jeunes médecins pour l'application des mesures strictes d'asepsie.

## Conflits d’intérêts

Les auteurs ne déclarent aucun conflit d'intérêts.
